# Prevalence of HIV and Other Sexually Transmitted Infections among Female Sex Workers in Kisumu, Western Kenya, 1997 and 2008

**DOI:** 10.1371/journal.pone.0054953

**Published:** 2013-01-23

**Authors:** Hilde M. Vandenhoudt, Lilian Langat, Joris Menten, Fredrick Odongo, Simon Oswago, Geoffrey Luttah, Clement Zeh, Tania Crucitti, Kayla Laserson, John Vulule, Anne Buve

**Affiliations:** 1 Institute of Tropical Medicine, Antwerp, Belgium; 2 Kenya Medical Research Institute, Kisian, Kenya; 3 Family Health Options, Kisumu, Kenya; 4 Centers for Disease Control and Prevention, Kisian, Kenya; Indiana University and Moi University, United States of America

## Abstract

**Background:**

In 1997, a survey in Kisumu found a prevalence of HIV infection among female sex workers (FSW) of 75%. Only 50% reported using a condom with the last client. In 2008, we conducted another survey to collect data to inform an intervention targeting FSW in Kisumu.

**Methods:**

In 2008 FSW were recruited by respondent-driven sampling. Women completed a questionnaire and were tested for HIV and other sexually transmitted infections (STIs). Multiple logistic regression analysis was done to explore factors associated with HIV-infection, and with condom use. Prevalence of HIV infection was compared in the two surveys from 1997 and 2008. Multivariate analysis was used to assess whether a change in HIV prevalence between the two surveys could be explained by changes in socio-demographic characteristics and/or behavioral factors.

**Results:**

481 FSW participated in the 2008 study. HIV prevalence was 56.5% (95% CI 52.0–61.6). Factors independently associated with HIV were age older than 29 years; being a widow; STI treatment in the past year; herpes simplex virus Type-2 infection; bacterial vaginosis; and trichomoniasis. Condom use with last client was reported by 75.0% (95% CI 70.9–78.9). Predictors of condom use with the last client were age older than 29 years; higher price paid by last client; ever having been tested for HIV. Predictors of unprotected sex were being drunk during last sex act; usually having sex during menses; and STI treatment in the past year. The odds ratio of HIV infection associated with year of survey was 0.49 (95% CI 0.33–0.75) after adjusting for socio-demographic and behavioral factors.

**Conclusions:**

The prevalence of HIV among FSW in Kisumu was found to be lower in 2008 than in 1997, while reported condom use was higher. However, access to HIV/STI prevention and care services needs to improve to further decrease HIV transmission between FSW and their clients.

## Introduction

Studies conducted in Nairobi, Kenya, in the 1980s and 1990s were among the first to draw attention to the important role female sex workers (FSW) and their clients played in the rapid spread of HIV infection in sub-Saharan Africa [1]–[6]. Work with mathematical models suggests that the role of FSW and their clients diminishes as the HIV epidemic becomes generalized as is nowadays the case in most countries in sub-Saharan Africa [7]. However, there is evidence that even in generalized epidemics a sizeable proportion of new HIV infections occur within the context of sex work. Using the UNAIDS Modes of Transmission Model it was estimated that in 2008, 14% of new infections in Kenya were attributable to FSW, their clients and the partners of clients [8]. The same exercise was conducted in Uganda, Mozambique and Malawi where it was estimated that 10%, 19% and 20% of new infections were attributable to commercial sex work [9]–[11]. An analysis of data from recent demographic health surveys from five African countries found that the proportion of HIV infections in adult men that can be attributed to sex with sex workers, ranged from 1.3% in Rwanda to 9.4% in Malawi [12]. Interventions targeting FSW and their clients therefore remain an important prevention strategy.

Nyanza Province is the region in Kenya worst affected by the HIV epidemic with HIV rates twice as high as the national HIV-prevalence [13]. In 1997, a cross-sectional survey was conducted among a sample of 300 self-acknowledged FSW who were recruited at their places of work in Kisumu, the capital of Nyanza Province [14]. HIV prevalence among these women was 75% and reported condom use with the last client was 50%. In 2006 a clinic was set up for FSW and a peer-led outreach program was initiated with the aim to reduce the transmission of HIV infection and other sexually transmitted infections (STIs) among FSW, their partners and clients in Kisumu district. The intervention is called ‘Pambazuko’(dawn or ‘the start of a new day’ in Kiswahili) and consists of HIV counseling and testing; treatment of STIs, condom and lubricant promotion and provision; contraceptives services; HIV care, including anti-retroviral treatment (ART); and behavioral change counseling.

In this paper, we report results of a cross-sectional survey that was conducted between October and December 2008. The objective of this survey was to estimate the prevalence of HIV, STI and condom use among FSW and the coverage of the Pambazuko services. In addition, we compare the findings of this survey with the results of the 1997 survey to assess whether there had been any changes in the prevalence of HIV and STIs and in reported condom use over the past ten years. The methods and the results of the 1997 survey have been published in 2001 [14].

## Methods

### Study Setting and Population

The study was conducted in Kisumu, the third largest town in Kenya with an approximate population of 450 000. Preceding the survey, we mapped venues where FSW solicit clients and conducted a capture-recapture exercise. This gave us an estimate of the FSW population in Kisumu in 2008 of 1692 (95% confidence interval (CI): 1578–1821) [15].

### Survey Conducted in 2008

#### Sampling

FSW were recruited through respondent-driven sampling (RDS), a sampling method that has been specifically developed for populations that are ‘hidden’ or difficult to reach [16]. Fifteen ‘seeds’ were selected among Pambazuko peer educators, representing all neighborhoods of Kisumu, with the distribution of seeds proportional to the numbers of FSW working in the different neighborhoods as estimated in the capture-recapture study [15]. After study participation, each seed received three referral coupons to recruit other participants. These participants in turn became recruiters who identified potential participants and provided them with a referral coupon for the Pambazuko clinic. Recruitment continued until the required sample size of 480 women was achieved. As it was expected that FSW working in the different neighborhoods may represent distinct and relatively isolated geographical subpopulations the maximum number of waves starting from a single seed was six, in order to avoid oversampling of certain neighborhoods.

#### Study procedures at the Pambazuko Clinic

At the clinic, study staff verified the validity of the coupon and screened women for study eligibility. Written consent was obtained in the language women felt most comfortable with. A detailed description of the consent process is given under the paragraph entitled “Ethics statement”. Legal age in Kenya is 18 years, but in this study, women aged 16 and 17 years were considered ‘mature minors’ and able to provide consent [17]. Participants used Audio Computer Assisted Self Interview (ACASI) to complete a questionnaire on socio-demographic characteristics; alcohol and substance use; reproductive health; sexual behavior; and HIV. After the interview, a clinical officer conducted a health assessment, including a full gynecological examination. A blood sample was taken for testing for HIV, syphilis, and herpes simplex virus type-2 (HSV-2); two endocervical swabs were taken for the diagnosis of *Chlamydia trachomatis* (CT) and *Neisseria gonorrhoeae* (NG) and one vaginal swab was collected from the posterior fornix for the detection of *Trichomonas vaginalis (*TV) and bacterial vaginosis (BV). Genital ulcerations found on examination were swabbed for etiological diagnosis. Suspected STIs and common ailments were treated according to Kenyan national guidelines. Participants received free male condoms and a tube of water-based lubricant. All participants received an appointment to collect their HIV and STI laboratory test results. However, those who wanted to learn their HIV status at the time of the study visit were referred to the counseling and testing service of the clinic.

Finally, the study coordinator explained the recruitment process, recorded data on the social network of the FSW, issued referral coupons and provided transport reimbursement equivalent to 4 US $. Participants were invited to return after two weeks to check if recruitees had presented themselves at the clinic. For each recruitee enrolled in the study, an additional incentive of 1.25 US $ was given to the study participants.

#### Laboratory procedures

Antibodies against HIV were detected using two standard ELISA tests in parallel, Vironostika (HIV Uniform II plus O kit; Organon Teknika, Boxtel, the Netherlands) and Enzygnost (Dade Behring, Marburg, GmbH, Germany). Concordant results were final. In case of discordance, a third ELISA, Genetic Systems rLAV EIA (Bio-Rad Laboratories, Redmond, WA, USA) was used as the tie breaker. For the detection of syphilis, serum was first tested with the BD-Macro-Vue RPR Card test kit (Becton-Dickinson, Sparks, MD, USA). Reactive sera were subsequently tested with the SERODIA-TPPA kit (Fujirebio Inc., Tokyo, Japan). Women with a positive RPR and TPPA were considered to have active, untreated syphilis. Antibodies against HSV-2 were detected with an HSV-2 type-specific IgG ELISA (Kalon Biological Ltd, Surrey, United Kingdom). Samples giving an optical density (OD) less than the cut-off×0.9 were considered as negative, while samples with an OD greater than the cut-off×1.1 were considered as positive. Samples giving an OD between cut-off×0.9 and cut-off×1.1 were considered as indeterminate.

The endocervical samples were first tested with the Amplicor CT/GC test (Roche Diagnostic Systems, Indianapolis, IN, USA) and all positive samples were confirmed with the BD ProbeTec assay™ ET *Chlamydia trachomatis* (CT) and *Neisseria gonorrhoeae* (GC) amplified DNA assay (Becton-Dickinson, Sparks, MD, USA) at the HIV/STI reference laboratory of ITM (Antwerp, Belgium). Samples with discordant results were re-tested with an in-house PCR.

The vaginal swab was rolled on a microscope slide, heat-fixed and stained with Gram’s stain. Scoring according to Nugent was performed for the diagnosis of BV. Thereafter the same swab was inoculated in a culture device for TV (Inpouch™, Biomed Diagnostics, San José, California, USA), incubated at room temperature and read on a daily basis with last reading at day three.

The etiological diagnosis of genital ulcerations was determined at ITM with an in-house multiplex PCR for the detection of *Treponema pallidum*, *Haemophylus ducreyi,* HSV-1 and HSV-2 [18].

### Methods of the Survey Conducted in 1997

The methods used in 1997 have been published in 2001 [14]. Prior to the survey a mapping exercise was conducted in Kisumu listing all places where FSW recruit their clients and number of FSW found in each location. From this list of locations and numbers of FSW, a random selection was made of locations. All women present at these locations at the time of the visit by the research team were approached and asked to participate in the study. Women who were willing to participate were invited to a clinic where informed consent was obtained and were the study procedures were conducted. The women were interviewed face-to-face with a questionnaire that served as basis for the questionnaire used in 2008 (the questionnaire that was used in 2008 was much longer). After the interview samples were taken for testing for HIV and other STIs.

Sera were tested for antibodies against HIV by ELISA (ICE HIV-1.0.2; Murex Diagnostics, Dartford, UK; or HIV-1/HIV-2 3rd Generation Plus; Abbott Laboratories, Abbott Park, Illinois, USA), and confirmed with a rapid test (Capillus HIV-1/HIV-2; Cambridge Diagnostics, Galway, Ireland; or HIV Multispot; Sanofi Diagnostics Pasteur S.A., Marne La Coquette, France). Antibodies against HSV-2 were detected with the Gull test (Gull Laboratories, Inc. Bad Homburg, Germany) which has since been withdrawn from the market. The specificities of the Gull test and of the Kalon test (Kalon Biological, Ltd. Surrey, United Kingdom) were similar but the Kalon test was more sensitive than the Gull test (92.3% vs 86.3%) [19]. Tests for syphilis and TV were the same as in 2008. In 1997 urine samples were used for the diagnosis of NG and CT. Samples were first tested with the Amplicor *Chlamydia trachomatis/Neisseria gonorrhea* test (Roche Diagnostics, Branchburg, New Jersey, USA) and the positive samples were confirmed with the LCx ™ *Neisseria* gonorrhea Assay or the LCx ™ *Chlamydia trachomatis* Assay (Abbott Laboratories). The prevalence rates for gonorrhea are thus not comparable in the two surveys: testing urine samples for NG has a lower sensitivity (50%) than endocervical samples while the LCx ™ has a lower specificity for NG than the BD ProbeTec assay ™ ET used in 2008.

### Data Management and Analysis

The analyses were performed with Stata 10.0 [20] and IBM SPSS Statistics version 19. Unweighted and weighted prevalence rates of HIV, STIs and condom use and 95% confidence intervals were calculated for 2008 [21]. The weighted rates took into account clustering in respondent driven sampling using RDSAT 6.0 software. We assessed if the RDS reached equilibrium by examining proportions of participants by age, education level and outcome categories across waves.

Factors associated with HIV and predictors of condom use in 2008 were assessed by multiple logistic regression. This analysis was performed using unweighted data as our main interest lies in determining individual risk factors and not population based associations and associations are unlikely to be influenced by moderate differences in selection probabilities [22], [23]. Covariates adjusted for age group (<20 years, 20–29 years, >29 years) with a p-value of <0.10 in bivariate analysis were included in model building, using a hierarchical approach [24]. In the HIV model, socio-demographic characteristics were included first, followed by behavioral factors and variables related to health; and finally STIs. To examine predictors of condom use, the following covariates were sequentially added to the model: socio-demographic variables, sexual behavior, HIV-testing and STI history, and exposure to Pambazuko services. P-values for associations were calculated using the likelihood ratio test. Covariates that were significantly associated with the outcome of interest within their level (p-value <0.05) remained in the final model even if no longer significant when adding predictors from a higher level in the hierarchical model building. This ensures that estimates remain corrected for possible lower level confounders. Interactions were tested within each level of the model building hierarchy.

In order to assess whether there was any change in HIV prevalence between 1997 and 2008, the datasets from the two surveys were merged. Only those variables were retained in the merged dataset that were collected in the same way, i.e. using the same questions in both surveys. The unweighted prevalence of HIV was compared in 1997 and 2008, by calculating the odds ratio for HIV associated with year of survey. Multivariate analysis was used to explore whether any change in HIV prevalence between 1997 and 2008 could be explained by changes in socio-demographic and behavioral factors among FSW. Socio-demographic and behavioral factors that were associated with HIV infection with a p-value <0.10 in bivariate analysis were entered in a multivariate model.

### Ethics Statement

For the 2008 survey the protocol, the informed consent documents and the questionnaires, and any subsequent modifications were reviewed by the CDC Institutional Review Board; the KEMRI Ethical Review Committee (ERC); the ITM Institutional Review Board, as well as the Ethical Committee of the University Teaching Hospital of Antwerp (Belgium). Explicit approval was also obtained for shipment of specimens to Belgium.

Informed consent was sought at the Pambazuko clinic after thorough explanation of study procedures and potential implications of study participation, including the expectation that participants would learn their HIV status. The consenting was conducted in the language with which the participant was most comfortable (Dholuo, Kiswahili or English). Participants signed or thumbprinted the consent form. Separate consent was asked for storage of samples.

Personal identifier information, risk behavior, and laboratory test results were recorded on separate forms. All data forms and specimens were linked through a unique barcode printed on stickers that were scanned into a database. Consent forms with personal identifiers were kept separately under lock. Names of participants were not collected. After completion of analyses, the data file linking identification with study ID number was destroyed.

## Results

### Survey Conducted in 2008

Of all referral coupons issued, 55% resulted in a completed clinic visit. Average network sizes, defined as the number of FSW known by name and met in the past month, were similar across age, education level and outcome categories. There were no significant differences between recruitment waves regarding participant characteristics or outcomes. We observed some preferential recruitment (homophily) within neighborhoods of Kisumu where FSW reported to primarily solicit clients, but no preferential recruitment or differences in network sizes within subgroups based on study outcomes, i.e. HIV infection and condom use. In total, 540 women reported at the clinic for study participation of whom 44 were not eligible. Of the latter women 28 denied that they engaged in sex work; 3 were under the age of 16; for 8 the coupon had expired or was not genuine; and 5 were unable to give informed consent. All 496 eligible women consented to participate in the study. After excluding the 15 seeds, data on 481 FSW were available for analysis. However HIV and STI results were missing for two participants who refused to provide samples.

Table 1 presents the socio-demographic and behavioral characteristics of the FSW. The median age of the participants was 26 years (interquartile range (IQR) 20–30) and 51% completed at least primary school. More than half (56%) of the women were either divorced or widowed and for 82%, sex work was the sole source of income. Most FSW (83%) had children, who usually lived elsewhere. At the time of the interview, the vast majority (89%) had been living in Kisumu for two years or longer.

**Table 1:** Socio-demographic and behavioural characteristics of FSW study participants in 2008 and in 1997.

	2008 survey		1997 survey	
	N = 481		N = 300	
A. Socio-demographic characteristics				
	N	%	N	%
**Age group**				
<20 years	35	7.3	33	11.0
20–29 years	308	64.1	186	62.0
>29 years	138	28.7	81	27.0
**Education** *				
Never went to school/Did not complete primary education	237	49.3	161	53.7
Did complete primary education	137	28.4	104	34.7
Secondary or higher education	107	22.3	35	11.7
**Marital status** **				
Never married	208	43.2	113	37.7
Currently married	5	1.0	6	2.0
Divorced/separated	146	30.4	155	51.7
Widowed	122	25.4	26	8.7
**Ethnic group** **				
Luo	407	84.6	196	65.3
**Religion**				
Anglican	113	23.5		
Catholic	208	43.2		
Other Christian	80	16.6		
Other	80	16.6		
**Place of birth**				
Kisumu	228	47.4		
Nyanza province	105	21.8		
Elsewhere	148	30.8		
**Current residence**				
Kisumu	399	83.0		
Village outside Kisumu	77	16.0		
Elsewhere	5	1.0		
**Number of years living in Kisumu** **				
<2 years	53	11.1	111	37.0
2–5 years	136	28.5	84	28.0
= >5 years	289	60.5	105	35.0
**Earns most income from sex work** **				
Yes	470	97.7	250	83.3
No	11	2.3	50	16.7
**B. Reproductive and Sexual history**				
**Has ever been pregnant**				
Yes	425	88.4		
No	56	11.6		
**Contraceptive use in the past year**				
None	130	27.0		
Pill	37	7.7		
Depo-provera	218	45.3		
Condom	72	15.0		
Sterilization	10	2.1		
Other method	14	2.9		
**Number of years doing sex work** **				
<5 years	211	48.0	228	76.5
5–9 years	153	34.8	45	15.1
>9 years	76	17.3	25	8.4
**Number of clients past week** **				
<3	146	30.4	239	80.2
3–4	170	35.3	41	13.8
>4	165	34.3	18	6.0
**Condom used with last client** **				
Yes	363	75.5	144	49.8
No	118	24.5	145	50.2
**Type of sex with last client** *				
Vaginal	258	59.5		
Oral	20	4.6		
Anal	72	16.6		
Combination	84	19.3		
**Price paid by last client**				
<100 Ksh (<1.3 US $)	56	11.6		
100–499 Ksh (1.3–6.7 US$)	223	46.3		
>499 Ksh (>6.7 US $)	202	42.0		
**FSW drunk during sex with last client**				
Yes	237	49.3		
No	244	50.7		
**Has at least one steady boyfriend** **				
Yes	374	77.7	264	90.4
No	107	22.2	28	9.6
**Condom use last time sex with boyfriend**				
Yes	179	48.0		
No	167	44.8		
Can’t remember	27	7.2		
**Usually has sex during menses** *				
Yes	154	32.0	72	24.4
No	327	68.0	223	75.6
**Usually washes vagina after sex**				
No	55	11.4		
Only water	76	15.8		
Water and soap	299	62.2		
Other disinfectant	51	10.6		
**Usually puts a product in vagina before sex** **				
Yes	160	33.3	5	1.7
None	321	66.7	294	98.3
**Ever raped as a FSW**				
Yes	193	40.1		
No	288	59.9		
**C. Other Risk Factors for HIV acquisition**				
**Blood transfusion during past 10 years**				
Yes	55	11.4		
No	426	88.6		
**Scarification in past 10 years**				
Yes	158	32.9		
No	323	67.1		
**Ever Intravenous Drug Use**				
Yes	35	7.3		
No	446	92.7		
**Injection past 12 months**				
Yes	384	79.8		
No	97	20.2		
**D. STI and HIV**				
**Treated for STI past year**				
Yes	89	18.5		
No	392	81.5		
**Ever tested for HIV**				
Yes	359	74.6		
No	122	25.4		

* Difference between two surveys statistically significant: p<0.05.

** Difference between two surveys statistically significant: p<0.001.

The median age of sexual debut and of initiation of sex work was 15 (IQR 14–17) and 20 years (IQR 17–25) respectively. The median duration of being engaged in sex work was 5 years (IQR 3–10). The median number of clients in the past working week was 3 (IQR 2–5) and 78% of women reported to have at least one steady boyfriend.

Of all participants, 75% had ever been tested for HIV, the majority (62%) in the previous year. Somewhat more than half of the participants (55%) were familiar with Pambazuko services, and 44% reported having sought services at the clinic.

HIV-prevalence was 56.5%; 83.8% of FSW tested positive for HSV-2; and 3.4% were diagnosed with active syphilis. Prevalence of NG and CT, TV and BV was 5.9%, 3.4%, 13.6% and 27.0% respectively (Table 2). Twenty three participants (5.6%) presented with genital ulcers. One specimen showed inhibition of the amplification assay. Nine of the 22 specimens were found to be positive for HSV-2, while no etiology could be established for the remaining 13. Condom use with the last client was reported by 75.0% of FSW, and 46.1% reported condom use during last sex act with their boyfriend.

**Table 2:** Prevalence estimates of HIV and other STI among FSW in 2008 and in 1997.

	2008 survey			1997 survey	
	n/N	Unweighted	Weighted %	n/N	%
		%	[95% CI]‡		
**HIV +**	277/479	57.8	56.5	221/296	74.7
			[52.0–61.6]		
**HSV-2**					
Positive	404/479	84.3	83.8	267/286	93.4
			[80.6–87.0]		
Negative	51/479			19/286	
Indeterminate	24/479				
**Syphilis +**	16/479	3.3	3.4	32/296	10.8
			[1.8–5.2]		
**Bacterial vaginosis (BV)**					
Positive	117/460	25.4	27.0		
			[22.2–31.4]		
Intermediary Nugent score	48/460				
Negative	295/460				
***T. vaginalis***					
Positive	67/475	14.1	13.6	117/259	45.2
			[10.6–16.7]		
***N. gonorrhoeae***					
Positive	26/474	5.5	5.9	39/296	13.2
			[3.8–8.1]		
***C. trachomatis***					
Positive	17/474	3.6	3.4	25/296	8.5
			[1.9–5.3]		

‡ Weighted for respondent driven sampling using RDS-analysis tool.

Table 3 presents factors associated with HIV infection. In the age adjusted analysis, marital status, ethnic group (Luo vs other), number of years doing sex work, money received for last sex act, STI treatment in the past year, HSV-2 infection, CT, TV and BV were all associated with HIV at the 0.10 significance level. In the model without STIs (data not shown) only age, marital status and price paid by last client remained significantly associated with HIV infection. Age older than 29 years (aOR 3.1; 95% CI: 1.1–8.4); being a widow (aOR: 2.5; 95% CI: 1.3–4.9); having received STI treatment in the past year (aOR: 2.3; 95% CI: 1.2–4.1); testing positive for HSV-2 (aOR: 7.0; 95% CI: 2.9–16.7); BV (aOR: 2.3; 95% CI: 1.3–3.9); TV (aOR: 2.4; 95% CI: 1.2–4.8) remained independently associated with HIV when adding STIs to the model (Table 3). Price received for last sex was no longer significantly associated.

**Table 3:** Factors associated with HIV in the 2008 survey (N = 479): Age group adjusted and multivariate analysis.

	HIV+	HIV+	Age adjusted OR(95% CI)†	P-value†	Multivariate aOR (95% CI)‡	P-value‡
	n/N	%	N = 479†		N = 394	
**Age group**						
<20 years	11/35	31.4	1		1	
20–29 years	132/306	52.9	2.4 [1.2–5.2]	**0.02**	1.5 [0.6–3.8]	0.34
>29 years	104/138	75.4	6.7 [3.0–15.0]	**<0.001**	**3.1 [1.1–8.4]**	**0.03**
**Education**						
Never went to school	76/117	65.0	1			
Did not complete primary education	68/118	57.6	0.8 [0.5–1.4]	0.43		
Did complete primary education	76/137	55.5	0.7 [0.4–1.2]	0.25		
Secondary or Higher education	57/107	53.3	0.6 [0.3–1.0]	0.06		
**Marital status**						
Never married	97/207	46.9	1		1	
Currently married/Divorced	86/151	57.0	1.2 [0.8–1.9]	0.33	1.1 [0.6–1.9]	0.73
Widowed	94/121	77.7	2.8 [1.6–4.8]	<0.001	**2.5 [1.3–4.9]**	**0.005**
**Ethnic group**						
Other	36/74	48.7	1			
Luo	241/405	59.5	1.6 [1.0–2.7]	0.06		
**Religion**						
Anglican	70/113	62.0	1			
Catholic	124/207	59.9	1.0 [0.6–1.6]	0.89		
Other Christian	41/80	51.3	0.7 [0.4–1.2]	0.18		
Other	42/79	53.2	0.7 [0.4–1.3]	0.26		
**Place of birth**						
Kisumu	124/227	54.6	1			
Nyanza province	60/104	57.7	1.0 [0.6–1.6]	0.98		
Elsewhere	93/148	62.8	1.3 [0.8–2.0]	0.30		
**Current residence**						
Kisumu	229/398	57.5	0.9 [0.5–1.5]	0.63		
Elsewhere	48/81	59.3	1			
**Number of years living in current place** *						
<2 years	30/53	56.6	1			
2–5 years	103/187	55.1	0.9 [0.5–1.8]	0.87		
>5 years	140/231	60.6	1.0 [0.5–1.8]	0.96		
**Has another job besides sex work**				0.41		
Yes	48/88	54.6	0.8 [0.5–1.3]			
No	229/391	58.6	1			
**Has ever been pregnant**						
Yes	245/423	57.9	0.8 [0.4–1.5]	0.50		
No	32/56	57.1	1			
**Contraceptives during past year**						
Hormonal contraceptives	142/254	55.9	1.0 [0.6–1.5]	0.92		
Other contraceptives	63/96	65.6	1.3 [0.7–2.2]	0.43		
No	72/129	55.8	1			
**Number of years doing sex work** *						
For each additional year			1.0 [1.0–1.1]	0.08		
<5 years	96/201	47.8	1			
5–9 years	94/147	64.0	1.6 [1.0–2.5]	0.05		
>9 years	59/86	68.6	1.4 [0.7–2.5]	0.31		
**Number of clients past week**						
For each additional client			1.0 [1.0–1.0]	0.60		
<3	91/146	62.3	1			
3–4	99/169	58.6	0.8 [0.5–1.4]	0.50		
>4	87/164	53.1	0.7 [0.4–1.1]	0.13		
**Type of sex with last client** *						
Vaginal	149/256	58.2	1			
Oral	15/20	75.0	2.1 [0.7–6.2]	0.18		
Anal	42/72	58.3	1.1 [0.6–1.8]	0.82		
Combination	51/84	60.7	1.2 [0.7–2.0]	0.58		
**Condom use with last client**						
Yes	210/361	58.2	0.9 [0.6–1.4]	0.78		
No	67/118	56.8	1			
**Price paid by last client**						
<100 Ksh (<1.3 US $)	39/56	69.6	1		1	
100–499 Ksh (1.3–6.7 US$)	130/222	58.6	0.6 [0.3–1.1]	**0.09**	0.6 [0.3–1.4]	0.24
>499 Ksh (>6.7 US $)	108/201	53.7	0.5 [0.2–0.9]	**0.02**	0.4 [0.2–1.1]	0.07
**FSW drunk during sex with last client**						
Yes	138/237	58.2	1.0 [0.7–1.4]	0.98		
No	139/242	57.4	1			
**Has at least one steady boyfriend**						
Yes	216/374	57.8	1.1 [0.7–1.8]	0.60		
No	61/105	58.1	1			
**Usually has sex during menses**						
Yes	89/154	57.8	1.1 [0.7–1.6]	0.76		
No	188/325	57.9	1			
**Usually washes vagina after sex**						
With soap/disinfectant	209/349	59.9	1.4 [0.9–2.1]	0.14		
With water/not	68/130	52.3	1			
**Usually puts a product in vagina before sex**						
Herbs/disinfectant/oil based lubricant	21/33	63.6	1.5 [0.7–3.2]	0.29		
Water based lubricant/female condom/none	256/446	57.4	1			
**Ever experienced rape as a FSW**						
Yes	109/193	56.5	0.9 [0.6–1.3]	0.48		
No	168/286	58.7	1			
**Blood transfusion during past 10 years**						
Yes	33/54	61.1	1.2 [0.6–2.2]	0.57		
No	244/425	57.4	1			
**Scarification past 10 years**						
Yes	95/158	60.1	1.1 [0.8–1.7]	0.52		
No	182/321	56.7	1			
**Ever Intravenous Drug Use**						
Yes	20/35	57.1	1.1 [0.5–2.3]	0.78		
No	257/444	57.9	1			
**Injection past 12 months**						
Yes	228/382	59.7	1.4 [0.9–2.2]	0.14		
No	49/97	50.5	1			
**Ever tested for HIV**						
Yes	210/357	58.8	1.1 [0.7–1.7]	0.57		
No	67/122	54.9	1			
**Treated for STI past 12 months**						
Yes	62/89	69.7	1.9 [1.**2**–**3**.2]	**0.01**	**2.3 [1.2**–**4.1]**	**0.01**
No	215/390	55.1	1			
**HSV-2** *						
Positive	259/404	64.1	7.8 [3.6–17.5]	**<0.001**	**7.0 [2.9**–**16.7]**	**<0.001**
Negative	8/51	15.7	1			
**Syphilis**						
Positive	8/16	50.0	0.7 [0.3–2.0]	0.50		
Negative	269/463	58.1	1			
**Bacterial Vaginosis** *						
Positive (Nugent score >6)	77/117	65.8	1.6 [1.0–2.5]	**0.04**	**2.3 [1.3**–**3.9]**	**0.003**
Negative	188/343	54.8	1		**1**	
***Trichomonas vaginalis*** *						
Positive	47/67	70.15	2.2 [1.2–3.9]	**0.01**	**2.4 [1.2**–**4.8]**	**0.02**
Negative	226/408	55.4	1		1	
***Neisseriae gonorrhoeae*** *						
Positive	14/26	53.85	0.9 [0.4–2.0]	0.81		
Negative	258/448	57.8	1			
***Chlamydia trachomatis*** *						
Positive	4/17	23.5	0.3 [0.1–1.0]	**0.04**		
Negative	268/457	58.6	1			

* variables with missing values; aOR (95% CI) Adjusted Odds Ratio (95% confidence interval);

† bivariate model adjusted for age group.

‡ multiple logistic regression model including STI (retained only participants with complete datasets).

Table 4 presents predictors of condom use with the last client. In the age-adjusted analysis, education, current residence (Kisumu vs elsewhere), price paid by last client, being drunk during last sex act, usually having sex during menses, ever been tested for HIV, reporting a previous positive HIV test result, being on ART, treatment for STIs in the past year, knowing Pambazuko and ever having visited Pambazuko clinic were associated with reported condom use (p-value <0.10). In the multiple logistic regression analysis, age older than 29 years (aOR: 2.5; 95% CI: 1.1–5.9), having received ≥500 KSh from last client (aOR: 2.5; 95% CI: 1.2–5.1), and ever having been tested for HIV (aOR: 2.2; 95% CI: 1.4–3.7) were positively associated with condom use. Being drunk during last sex act (aOR: 0.5; 95% CI: 0.3–0.9), usually having sex during menses (aOR: 0.6; 95% CI: 0.4–0.9), and having received STI treatment in the past year (aOR: 0.5; 95% CI: 0.3–0.8) were independent predictors of lack of condom use.

**Table 4:** Predictors of Condom use with last client among FSW (N = 481) – Age-adjusted and multivariate analysis.

	Condom use		Age adjusted OR(95% CI)†	P-value†	MultivariateaOR(95% CI)‡	P-value‡
	n/N	%			N = 481	
**Age group**						
<20 years	21/35	60.0	1		1	
20–29 years	232/308	75.3	2.0 [1.0–4.2]	0.05	1.96 [0.89–4.31]	0.09
>29 years	110/138	79.7	2.6 [1.2–5.8]	0.02	**2.49 [1.06**–**5.85]**	**0.04**
**Education**						
Never went to school	87/117	74.4	1			
Did not complete primary education	81/120	67.5	0.75 [0.42–1.32]	0.32		
Did complete primary education	108/137	78.8	1.35 [0.75–2.42]	0.32		
Secondary or Higher education	87/107	81.3	1.4 [0.8–2.8]	0.23		
**Marital status**						
Never married	159/208	76.4	1			
Currently married/Divorced	114/151	75.5	0.8 [0.5–1.3]	0.38		
Widowed	90/122	73.8	0.67 [0.4–1.2]	0.16		
**Ethnic group**						
Other	59/74	79.7	1			
Luo	304/407	74.7	0.8 [0.4–1.4]	0.42		
**Religion**						
Anglican	84/113	74.3	1			
Catholic	156/208	75.0	1.1 [0.6–1.8]	0.83		
Other Christian	60/80	75.0	1.0 [0.5–2.0]	0.88		
Other	63/80	78.75	1.3 [0.6–2.6]	0.48		
**Place of birth**						
Kisumu	174/228	76.3	1			
Nyanza province	78/105	74.3	0.8 [0.5–1.4]	0.51		
Elsewhere	111/148	75.0	0.9 [0.5–1.4]	0.55		
**Current residence**						
Kisumu	308/399	77.2	1.6 [01.0–2.8]	0.06		
Elsewhere	55/82	67.1	1			
**Number of years living in current place** *						
<2 years	45/53	84.9	1			
2–5 years	141/189	74.6	0.5 [0.2–1.2]	0.13		
>5 years	173/231	74.9	0.5 [0.2–1.1]	0.09		
**Has another job besides sex work**						
Yes	71/89	79.8	1.3 [0.7–2.3]	0.32		
No	292/392	74.5	1			
**Has ever been pregnant**						
Yes	323/425	76.0	1.1 [0.6–2.1]	0.72		
No	40/56	71.4	1			
**Contraceptives during past year**						
Hormonal contraceptives	195/255	76.5	1.4[0.9–2.3]	0.14		
Other contraceptives	78/96	81.3	1.8 [0.9–3.4]	0.07		
No	90/130	69.2	1			
**Number of years doing sex work** *						
For each year of sex work			1.0 [0.9–1.0]	0.13		
<5 years	155/202	76.7	1			
5–9 years	110/147	74.8	0.7 [0.4–1.2]	0.25		
>9 years	67/86	77.9	0.7 [0.4–1.4]	0.33		
**Number of clients past working week**						
For each client during past week			1.0 [0.1–1.0]	0.77		
<3 clients	106/146	72.6	1			
3–4 clients	128/169	75.3	1.1 [0.7–1.9]	0.61		
>4 clients	129/164	78.2	1.4 [0.8–2.3]	0.24		
**Type of sex with last client** *						
Vaginal	205/258	79.5	1			
Oral	14/20	70.0	2.1 [0.7–6.1]	0.29		
Anal	51/72	76.2	1.1 [0.6–1.5]	0.15		
Combination	64/84	77.0	1.2 [0.7–2.0]	0.54		
**Price paid by last client**						
<100 Ksh (<1.3 US $)	37/56	66.1	1		1	
100–499 Ksh (1.3–6.7 US$)	154/223	69.1	1.1 [0.6–2.1]	0.70	1.0 [0.5–1.9]	0.99
>499 Ksh (>6.7 US $)	172/202	85.2	2.9 [1.4–5.7]	0.002	**2.5 [1.2–5.1]**	**0.01**
**FSW drunk during sex with last client**						
Yes	161/237	67.9	0.4 [0.3–0.7]	<0.001	**0.5 [0.3–0.9]**	**0.01**
No	202/244	82.8	1		1	
**Has at least one steady boyfriend**						
Yes	282/375	75.2	1.0 [0.6–1.6]	0.96		
No	81/106	76.4	1			
**Usually has sex during menses**						
Yes	101/154	65.6	0.5 [0.3–07]	0.001	**0.6 [0.4–0.9]**	**0.02**
No	262/327	80.1	1		1	
**Usually washes vagina after sex**						
With soap/disinfectant	270/350	77.1	1.4 [0.9–2.2]	0.16		
With water/not	93/131	70.1	1			
**Usually puts a product in vagina before sex**						
Herbs/disinfectant/oil based lubricant	22/33	66.7	0.7 [0.3–1.4]	0.29		
Water based lubricant/female condom/none	341/448	76.1	1			
**Ever experienced rape as a FSW**						
Yes	136/193	70.5	0.6 [0.4–1.0]	0.03		
No	227/288	78.8	1			
**Ever been tested for HIV**						
Yes	286/359	79.7	2.3 [1.5–3.6]	<0.001	**2.2 [1.4–3.7]**	**0.001**
No	77/122	63.1	1		1	
**Previous HIV test positive**						
Yes	128/156	82.1	1.3 [1.0–2.6]	0.06		
No	235/325	72.3	1			
**Reported to receive ART**						
Yes	97/115	84.4	1.98 [1.1–3.3]	0.03		
No	266/366	72.7	1			
**Received treatment for STI in past 1 year**						
Yes	57/89	64.0	0.5 [0.3–0.8]	0.01	**0.5 [0.3–0.8]**	**0.01**
No	306/392	78.1	1		1	
**Knows Pambazuko programme**						
Yes	231/264	80.7	1.8 [1.2–2.7]	0.01		
No	150/217	69.1	1			
**Has visited Pambazuko clinic**						
Yes	182/223	81.6	1.8 [1.2–2.8]	0.01		
No	181/259	70.2	1			

* variables with missing values; aOR (95% CI) Adjusted Odds Ratio (95% confidence interval);

† bivariate model adjusted for age group.

‡ multiple logistic regression model.

### Comparison between 1997 and 2008

Table 1 presents the socio-demographic and behavioral characteristics of the women in the 2008 survey and 1997 survey. The median age of the FSW who participated in the 1997 survey was slightly lower compared to the 2008 survey (25 years vs 26 years). In the 2008 survey more women had attended secondary school or higher education than in 1997. In the 2008 survey a higher proportion of women were widowed and a lower proportion divorced compared to 1997. Median age at first sex in exchange for money was lower in 2008 than in 1997 (20 years vs 22 years) and the median number of clients in the past working week was higher (3 vs 1). The proportion of FSW who reported at least one steady partner was lower in 2008 than in 1997 (78% vs 90%). Reported condom use with last client was higher in 2008 than in 1997.

The prevalence of HIV infection and of other STIs including syphilis, gonorrhea, chlamydial infection and trichomoniasis was lower in 2008 than in 1997(Table 2). The OR of HIV associated with year of survey was 0.47 (95% CI 0.34–0.64). This OR changed very little after adjusting for socio-demographic and behavioral characteristics (Table 5).

**Table 5:** Factors associated with HIV infection in FSW participating in the surveys of 1997 and 2008: bivariate and multivariate analyses.

	HIV positive		OR	p	aOR
	n/N	HIV %			
**Year of survey**					
1997	221/296	74.7	1	<0.001	1
2008	202/479	57.8	0.47		0.50
**Agegroup**					
<20	37/73	50.7	1	<0.001	1
20–24	152/273	55.7	1.22		1.27
25–29	151/217	69.6	2.23		2. 07
>29	158/212	74.5	2.85		2.02
**Education**					
Never went to school/primary education not completed	267/394	67.8	1	0.06	1
Primary education completed	162/259	62.5	0.79		0.71
Secondary or higher education	69/122	56.6	0.62		0.66
**Marital status**					
Never married	173/319	54.2	1	0.00	1
Currently married/Divorced/Separated	212/309	68.6	1.84		1.40
Widowed	113/147	76.9	2.81		2.51
**Ethnic group**					
Luo	391/599	65.3	1.21	0.28	
Other	107/176	60.8	1		
**Duration of residence in Kisumu**					
<2 years	115/164	70.1	1	0.19	
2–4 years	139/216	64.4	0.77		
= >5 years	243/392	62.0	0.70		
**Duration of sex work**					
<5 years	272/436	62.4	1	0.04	1
5–9 years	124/196	63.3	1.04		1.08
>9 years	76/100	76.0	1.91		1.55
**Earns most income from sex work**					
Yes	457/715	63.9	0.82	0.49	
No	41/60	68.3	1		
**Number of clients past week**					
<3	269/382	70.4	1	0.001	1
3–4	128/209	61.2	0.66		0.79
>4	99/182	54.4	0.50		0.69
**Condom used with last client**					
Yes	317/503	63.0	0.85	0.32	
No	174/261	66.7	1		
**Number of steady boyfriends**					
0	82/133	61.7	1	0.02	1
1	95/127	74.8	1.85		1.25
>1	313/507	61.7	1.00		0.93
**Usually puts product in the vagina before sexual intercourse**					
Yes	104/164	63.4	0.96	0.81	
No	393/610	64.4	1		
**Usually has sexual intercourse during menses**					
Yes	138/224	61.6	0.86	0.35	
No	356/546	65.2	1		

## Discussion

We found a high prevalence of HIV infection, 56.5%, among a sample of FSW in Kisumu and suboptimal levels of condom use. However, compared to the survey in 1997 the prevalence of HIV infection and of other STIs appeared to have decreased while the proportion of women reporting use of a condom with the last client increased from 50% to 75.0%. In both surveys study participants were recruited in the community but the sampling methods were somewhat different and we cannot exclude that differences in sampling methods may have introduced a bias in the comparison between the two surveys. However we were able to adjust for differences in socio-economic and behavioral characteristics and the OR for HIV associated with year of survey remained below 1, suggesting that the difference in HIV prevalence between the two survey years was not due to differences in socio-economic and behavioral characteristics. Several explanations for this decline in HIV can be put forward. In our analysis the OR of HIV associated with year of survey changed very little after adding socio-economic and behavioral variables to the model. This suggest that the decline in HIV cannot be explained by socio-economic and behavioral changes in the sex worker population in Kisumu, with the possible exception of condom use. The most likely explanation for the decrease in HIV prevalence is a decline in background HIV prevalence in Kisumu and an increase in condom use among FSW. HIV prevalence among antenatal clinic attendees in Kisumu was 32% in 1997 and 18% in 2006 suggesting a decline in HIV prevalence in the general population (Kenya Ministry of Health surveillance data). Condom use by FSW was self-reported and though we used ACASI, some social desirability bias cannot be excluded. However the condom use as reported by FSW in Kisumu was in line with the condom use as reported by FSW working along the Trans-Africa highway, 79% of whom reported using a condom with all contacts [25]. The increase in condom use by FSW in Kisumu is likely attributable to a general increased awareness of HIV in Western Kenya over the past ten years.

At the individual level reported condom use was not associated with a decreased risk of HIV infection in 2008 (adjusted OR of HIV infection associated with condom use with the last client: 0.9). A possible explanation for this finding can be found in the analysis of predictors of condom use. Women who had been tested for HIV reported higher condom use. This suggests that counseling, including counseling of HIV infected women, may have had a positive effect on condom use or, alternatively, led to social desirability bias in the responses to the questions about condom use.

The analysis of predictors of condom use identified a number of barriers to using condoms. Condoms were used less frequently with boyfriends than with clients (46.1% in last sex act vs 75.0%), a finding consistent with other studies [26]–[32] which highlights the need to address condom use within the context of different partnerships. In our study, 48% of FSW reported that they were drunk during sex with their last client and these women were less likely to use a condom. Alcohol abuse is an important impediment to consistent condom use and also to ART adherence [33]–[35]. It is therefore critical that interventions targeting FSW provide counseling on the link between alcohol and unprotected sex as well as address alcohol addiction among FSW. Lastly, lower price per sex act was independently associated with lower condom use. A lower price per sex act was also associated with an increased risk of HIV infection but the association was only statistically significant in Model 1 (without STIs). Previous studies have documented an association between low socio-economic status of FSW and HIV risk [36], [37]. There are however several possible mechanisms underlying this association and cross-sectional surveys do not allow us to establish whether HIV infection led to poverty and entry into sex work, or whether poverty led to sexual risk taking and HIV infection. In our study, a high proportion of FSW were divorced or widowed and widowhood was associated with an increased risk of HIV infection. Many of these women most likely became widows because their husband died of AIDS and were probably already HIV infected when they entered sex work as a survival strategy.

Treatment of an STI in the previous year, HSV-2 infection, BV and TV, were all independently associated with an increased risk of HIV infection. As this was a cross-sectional study, it is not possible to establish the temporal relationship between HSV-2 infection, BV and TV, and HIV infection. In HIV uninfected women, these STIs increase their risk of HIV acquisition; in HIV infected women, they increase the risk of onward HIV transmission. Syndromic management of curable STIs is an essential part of interventions targeting FSW and their clients, but so far less attention has been paid to the diagnosis and management of BV in FSW and more research needs to be done on how best to manage this reproductive tract infection.

Two years into Pambazuko implementation, less than half of FSW did seek services at the FHOK clinic and more work needs to be done to improving access to health care. Data on coverage were similar to findings from the capture-recapture exercise preceding the survey [15]. One way of increasing coverage is strengthening the outreach component with peer educators, as illustrated by the AVAHAN project in India [29]. Our study findings also confirm the need to address women’s vulnerability for effective HIV-prevention rather than focusing on risk behavior change alone. However, addressing vulnerability in combination with risk reduction remains a complex undertaking, and more research is needed to provide guidelines for effective implementation.
